# Impact of *TNF-α* Gene Polymorphisms on Pancreatic and Non-Small Cell Lung Cancer-Induced Cachexia in Adult Egyptian Patients: A Focus on Pathogenic Trajectories

**DOI:** 10.3389/fonc.2021.783231

**Published:** 2021-11-18

**Authors:** Rana Yehia, Mona Schaalan, Dalaal M. Abdallah, Amr S. Saad, Neven Sarhan, Samira Saleh

**Affiliations:** ^1^ Clinical Pharmacy and Pharmacy Practice, Faculty of Pharmacy, Misr International University, Cairo, Egypt; ^2^ Pharmacology and Toxicology Department, Faculty of Pharmacy, Cairo University, Cairo, Egypt; ^3^ Oncology Department, Faculty of Medicine, Ain Shams University, Cairo, Egypt

**Keywords:** pancreatic and NSCL cancer, cachexia, single-nucleotide polymorphism, *TNF-α* gene, *miR-155*, SOCS1, Foxp3, TAB2

## Abstract

**Background:**

Cachexia is a frequent syndrome in pancreatic and non-small cell lung (NSCL) cancer patients. The storm of cancer-induced inflammatory cytokines, in particular TNF-α, is a crucial pathogenic mechanism. Among the molecular alterations accused of cancer-induced cachexia, *TNF-α 308 G/A* (rs1800629) and *−1031T/C* (rs1799964) are single-nucleotide polymorphisms (SNPs) within the gene encoding this pro-inflammatory cytokine. Recent studies have demonstrated the crucial role of non-coding microRNAs (*miRNAs*) in pathogenesis of different diseases including cachexia. Moreover, the mechanistic cytokine signaling pathway of *miR-155*, as a *TNF-α* regulator, supports the involvement of SOCS1, TAB2, and Foxp3, which are direct targets of *TNF-α* gene.

**Aim:**

A case–control study (NCT04131478) was conducted primarily to determine the incidence of *TNF-α 308 G/A* (rs1800629) and *−1031T/C* (rs1799964) gene polymorphisms in adult Egyptian patients with local/advanced or metastatic pancreatic or NSCL cancer and investigate both as cachexia risk factors. The association of gene polymorphism with cachexia severity and the expression of *miR-155* in cachectic patients were analyzed. A mechanistic investigation of the cytokine signaling pathway, involving SOCS1, TAB2, and Foxp3, was also performed.

**Results:**

In both pancreatic and NSCL cancer cohorts, the mutant *TNF-α* variant of *308 G/A* was positively associated with cachexia; on the contrary, that of 1031T/C was negatively associated with cachexia in the NSCL cancer patients. *MiR-155* was higher in cachexia and in alignment with its severity in the cachectic group as compared with the non-cachectic group in both the pancreatic and NSCL cancer patients. Though TAB2 did not change to any significant extent in cachectic patients, the levels of SOCS1 and Foxp3 were significantly lower in the cachectic group as compared with the non-cachectic group.

**Conclusion:**

Carriers of the A allele *308 G/A* gene and high *miR-155* are at greater risk of cachexia in both the pancreatic and NSCL cancer patients; however, the mutant variant of *1031T/C* gene is protective against cachexia in the NSCL cancer patients. Finally, high levels of *miR-155* in the cachectic group lead to negative feedback inhibition of both SOCS1 and Foxp3 in both the pancreatic and NSCL cancer patients.

## Introduction

Cachexia is a devastating, multifactorial syndrome that is observed in the majority of end-stage cancer patients (1–3). It is more acute in certain incurable malignancies, such as pancreatic and NSCL cancers (4–6). The current understanding of cancer cachexia indicates that factors secreted by tumors together with factors secreted as a result of the tumor–host interaction initiate systemic inflammation and metabolic disturbances, which in turn trigger muscle wasting. Thus, systemic inflammation is thought to be a major mediator of cancer cachexia (7). TNF-α is probably the most characterized cytokine in cachexia, as it promotes anorexia and skeletal muscle wasting mainly through the NF-κB pathway (8). In a feedforward loop, this cytokine functions in controlling the transcription factor nuclear factor kappa light chain enhancer of activated B cells (NF-κB), which helps in adjusting immune and inflammatory responses and consequently leads to the generation of specific cytokines that have a role in proteolysis and breakdown of myofibrillar proteins (9). Accordingly, TNF-α-mediated NF-κB activation promotes wasting of muscle that leads to bodyweight loss ending with cancer cachexia (10).

In the last few years, several functional single-nucleotide polymorphisms (SNPs) within cytokines’ genes have been identified and described as cancer-related genetic alterations. The most important ones seem to be SNPs located within the promoter of *TNF-α* because of their ability to regulate gene expression and, consequently, the expression of the TNF-α protein. Among frequently investigated SNPs, the 308 G/A (rs1800629) and −238 G/A (rs361525) are potentially involved in tumor aggressiveness, prognosis, and risk of malnutrition (11, 12). Notably, there are only few data concerning the role of *TNF-α −1031T/C* SNP (rs1799964) in the regulation of systemic inflammatory response; however, the latest studies have demonstrated the role of this SNP as cachexia-related genetic alteration (13, 14). Accordingly, the significant role of the systemic inflammatory response mediated by TNF-α in the etiopathology of cachexia encourages investigating SNPs of *TNF-α* as cachexia-related risk factors.

MicroRNAs (miRNAs) represent another class of molecules that may be involved in muscle wasting (15). Altered expression of microRNAs has been shown to be involved in skeletal muscle homeostasis in health and disease (16–19). Moreover, several lines of evidence demonstrate that *miR-155* is overexpressed in a number of neoplastic diseases (20), where altered *miRNA* expression has been found in hematological malignancies, thyroid carcinoma breast and colon cancer (21); thus, it is considered to be a marker of poor prognosis (22). Additionally, many studies have highlighted the role of miRNAs in the pathophysiology of cancer cachexia (23) particularly *Mir-155* (24).

Noteworthy, Jiang et al. (25) identified SOCS1 as a novel target of *miR-155* in breast cancer cells (25). The suppressors of cytokine signaling (SOCS) protein family are described as direct regulators of janus kinase (JAK)/signal transducer and activator of transcription (STAT) signaling pathway in cancer. Overexpression of *SOCS* gene and consequently the SOCS proteins were observed in breast cancer; and a higher expression level was significantly associated with high-grade tumors (26). These data provide further evidence for the proto-oncogenic contribution of SOCS protein in cancer. Furthermore, the forkhead transcription factor is an immune regulator where the Foxp3 member is mainly expressed in CD4+ cells, which directly suppress the immune system through suppressing nuclear transcript abundant transcript 1 (NEAT1) and NF-κB and consequently repress interleukin-2 (IL-2) and T-cell cytokines (27). Recently, high expression levels of Foxp3, at genetic and protein levels, are significantly associated with tumor invasion in pancreatic ductal adenocarcinoma (PDAC) and lung adenocarcinoma (28). Moreover, transforming growth factor β binding activated kinase 1 protein 2 (TAB2) is an inflammatory mediator in cancer pathogenesis. Aberrant expression of TAB2 protein is significantly associated with cancer progression through activation of mitogen-activated protein kinase (MAPK) and NF-κB signaling pathway. A higher expression level of TAB2 was observed in ovarian cancer (29).

Genetic studies on cancer-associated cachexia remain highly controversial. Thus, the present study focused on two types of solid cancers with different pathological entities, pancreatic cancer “digestive system cancer” and lung cancer “respiratory system cancer.” Cumulative evidences have proven that TNF-α is a pro-cachectic protein; therefore, two SNPs of *TNF-α* gene, as well as *miR-155* expression, have been investigated and correlated the gene genotype with risk of cancer-associated cachexia. The selection of SNPs was based on the global and European minor allele frequency (MAF): *TNF-α 308 G/A* (rs1800629) and *−1031T/C* (rs1799964) SNP. Finally, the involvement of SOCS1, TAB2, and Foxp3 as direct targets for *TNF-α* gene in both cancer types was assessed.

## Patients and Methods

### Study Design and Population

This case–control study was conducted at the Oncology Department, Faculty of Medicine, Ain Shams University (Cairo, Egypt). Pancreatic and NSCL cancer patients (n = 203) were recruited in this study; their mean age was 51.45 ± 9.7 and ranged at 20–77 years. They were divided into two subgroups according to the degree of depletion of energy stores, body mass index (BMI), and ongoing weight loss; the first subgroup includes 94 patients who represent the comparative control non-cachectic group. The second subgroup includes 109 cachectic cancer patients who were then classified into pre-cachexia, cachexia, and refractory cachexia according to the cachexia severity index (30) by comparing their current weight with their actual one recorded on first admission. Pre-cachexia is defined as a ≤5% weight loss with anorexia and metabolic change; cachectic patients present with weight loss of >5%, BMI < 20, and weight loss of >2% or sarcopenia and weight loss of >2%; they also often had reduced food intake and systemic inflammation. In refractory cachexia, the cancer is pro-catabolic and not responsive to treatment (1). According to the type of cancer, patients were additionally classified into two groups: 71% of cases were subclassified into cachectic (n = 69) and non-cachectic (n = 76) groups who received *Xeloda* (pancreatic cancer; n = 145), and 29% were also subclassified into cachectic (n = 40) and non-cachectic (n = 18) groups treated with *Gem/Cis* (lung cancer).

### Approval and Ethical Considerations

The current study was approved by the Research Ethics Committee of Faculty of Pharmacy, Cairo University (Cairo, Egypt; PT-2387), as well as the Ethical Committee of the Oncology Department, Ain Shams University, and registered in ClinicalTrials.gov (trial registration number NCT04131478). The recruited patients provide the required informed consent.

### Inclusion and Exclusion Criteria

Inclusion criteria were based on a thorough history taking, and clinical and pathological examinations. Patients were considered eligible if they meet the following criteria: a medical diagnosis of cancer (e.g., lung or pancreatic), locally, advanced, or metastatic cancer scheduled for first-line cytotoxic chemotherapy; starting or continuing chemotherapy at the time of screening for participants where the duration was set based on standard period of first-line chemotherapy; and age between 18 and 80 years.

On the other hand, patients with the following criteria were excluded from the study: if they planned to have surgical procedures at the time of recruitment, have underwent surgery during the study or in the month prior to the study, and did not have chemotherapy scheduled post-surgery. Also, patients with comorbidities that could affect the interpretation of study findings were excluded, e.g., HIV, Alzheimer’s disease, movement disorder, acute myocardial infarction within the last 3 months, hepatitis, open burn sites or infected wounds, esophageal cancer with a swallowing difficulty in mechanical nature, or an uncorrected mechanical digestive obstruction. Pregnant, nursing women or patients with disorders associated with change in *miR-155* level (rheumatic arthritis, osteoarthritis, atopic eczema, Down’s syndrome, breast cancer, endometrioid adenocarcinoma, acute myeloid leukemia (AML), chronic lymphocytic leukemia (CLL), and papillary carcinoma thyroid tumors) were excluded too; and finally, patients with inflammatory and autoimmune diseases (multiple sclerosis, psoriasis, and systemic lupus erythematous) were excluded.

### Study Outcomes

*Primary outcomes*: to detect the incidence of *TNF-α 308 G/A* (rs1800629) and *−1031T/C* (rs1799964) gene polymorphism and investigate both as cachexia risk factors in local/advanced/metastatic pancreatic or NSCL cancer in adult Egyptian patients; to determine the association of gene polymorphism with cachexia severity; to assess the expression of *miR-155* in cachectic patients and its association with cachexia severity; and to verify the involvement of the cytokines SOCS1, TAB2, and Foxp3 signaling pathway in pancreatic and NSCL cancers.

*Secondary outcomes*: to measure the association between *TNF-α 308 G/A* (rs1800629) and *−1031T/C* (rs1799964) gene polymorphism in the selected patient groups and the development of cancer cachexia; and to analyze the correlation between *miR-155* gene expression and SOCS1, TAB2, or Foxp3 in cachectic pancreatic or NSCL cancer patients.

### Sample Collection and Genotyping Procedure

A venous blood sample (5 ml) was withdrawn from each participant under complete aseptic conditions and divided into two portions, as follows: 2 ml of blood was placed in an EDTA-containing tube for DNA extraction used for genotyping of the *TNF-α* gene polymorphism, and 3 ml of blood was left at room temperature for 30 min for spontaneous clotting, and then serum was separated by centrifugation at 3,000 rpm for 10 min. The serum samples were used for RNA extraction and for ELISA technique. Both samples were stored at −80°C until analysis.

### Clinicopathological Assessments

Blood urea nitrogen (BUN), alanine aminotransferase (ALT), aspartate aminotransferase (AST), total serum bilirubin, and direct serum bilirubin were analyzed by spectrophotometric assay on fully automated clinical chemistry analyzer (Synchron LX^®^ Systems; Beckman Coulter, CA, USA); and hemoglobin, platelets, and total leukocytic count are also measured by the AcT 5diff Cap Pierce hematology analyzer (Beckman Coulter hematology analyzer; CA, USA).

### *TNF-α* Gene Polymorphism by Pharmacogenetics Analysis/Genotyping

Genomic DNA was extracted from peripheral blood leukocytes using the automated QIAcube device (Qiagen, Hilden, Germany) according to the manufacturer’s guidelines. The selected polymorphisms were then genotyped by TaqMan allelic discrimination method according to the manufacturer’s recommendations (Applied Biosystems, Thermo Fisher Scientific, MA, USA).

### Amplification of *MiR-155* Using qRT-PCR Technique

Total *miRNA* was isolated from patients’ sera by using the “miRNeasySerum/Plasma Kit” (Qiagen). *MiR-155* was reversibly transcribed using miScript II RT Kit (Qiagen). In a reverse transcription reaction with miScript HiSpec Buffer, mature miRNAs are polyadenylated by poly(A) polymerase and converted into cDNA by reverse transcriptase with oligo-dT priming; and the cDNA was then used for real-time PCR quantification of mature *miRNA* expression. Relative miRNA expression levels for the candidate *miR-155* were analyzed by *mi*Script SYBR Green PCR Kit (Qiagen) and specific primers (Hs_*miR-155*_2 miScript Primer Assay [cat#: 218300], which target mature *miR-155* (cat#: MS00031486; Qiagen) and Hs_SNORD68_11 miScript Primer Assay cat#: 218300) as housekeeper gene (HK), which targets SNORD68 small nucleolar RNA, C/D box 68 (cat#: MS000337). The amplification was done using 5 Plex Rotor Gene RealTime PCR Analyzer (Qiagen). The relative quantitation of miR-155 was calculated using the equation 2^−ΔΔCt^ test control.

### Quantification of SOCS1, TAB2, and Foxp3 Serum Levels Using ELISA Technique

Serum *SOCS1* (cat#: SL3093Hu), *TAB2* (cat#: SL3094Hu), and *Foxp3* (cat#: SL2462Hu) were measured in patients’ sera using the corresponding human ELISA Kit (SunLong Biotech Co., Hangzhou, China).

### Statistical Analysis

The sample size was calculated using G* program, version 3.1.9.4, setting alpha error at 5% and power at 95% and the allocation ratio for N1/N2 = 1. So assuming an effect size of 0.5 (Cohen’s f) between two groups produced a total sample size of not less than 184 subjects. Parametric data were presented as mean ± SD and range, while non-parametric data were presented as median and range; categorical variables were given as numbers (percentage). Data were checked for normality using the Kolmogorov–Smirnov test and homogeneity with chi-squared test, as appropriate. For parametric data, the comparison between the two groups was done by Student’s *t*-test, whereas for multiple comparisons, *one-way ANOVA* followed by Bonferroni’s post-hoc test was performed. For non-parametric variables, the *Mann–Whitney* test was adopted to compare the two groups, whereas the *Wilcoxon Signed Rank* test assessed the statistical significance differences between two dependent samples. Logistic regression analysis was used for the association between two SNPs and susceptibility to cancer-associated cachexia and was given as odds ratios (ORs) and corresponding 95% CI. The *Hardy–Weinberg equilibrium (HWE)* and the association between *TNF-α* gene polymorphisms and risk of cancer-related cachexia were calculated by SNPstats online software (http://www.snpstats.net/start.htm), which assessed the frequency distribution between cachectic and non-cachectic Egyptian adult cancer patients of four genetic models: codominant, dominant, recessive, and overdominant (31). Spearman’s correlation analysis was used between *miR-155* gene expression and serum levels of SOCS1, TAB2, and Foxp3 in cachexia-related with pancreatic and NSCL cancers. The collected data were revised, coded, and tabulated using *SPSS version 24* (IL, USA). All graphs were plotted by *GraphPad Prism Software 8.4.2* (CA, USA). The level of significance is taken at a p-value of <0.05.

### Bioinformatics Analysis of *TNF-α* Gene

In order to infer interrelationships among the *TNF-α* gene and cancer cachexia, the SNPs, and *TNF-α* (rs1800629) and (rs1799964), selection was based on global and European MAF published on the National Center for Biotechnology Information in collaboration with the National Human Genome Research Institute (dbSNP) accessed from https://www.ncbi.nlm.nih.gov/snp. The frequency of selected SNPs was double-checked from Pharmacogenomics Knowledgebase (*Pharm GKB*) accessed from https://www.pharmgkb.org.

## Results

### Clinical and Biochemical Features of Pancreatic and Non-Small Cell Lung Cancer Patients

The current study was conducted on 203 adult Egyptian cancer patients who were sub-classified into two main groups: the cachectic group (n = 109) and non-cachectic group (n = 94) where their baseline characteristics are given in **Table 1**. There was no difference between the cachectic and non-cachectic patients regarding the mean of age, distribution of gender, presence, and number of comorbidities with the majority having only one comorbid disease; the only difference was the type of cancer/chemotherapy. Significant associations existed between cachexia and non-cachexia subgroups for BUN and TLC in the pancreatic cancer patients (**Table S1**) and direct serum bilirubin in the NSCL cancer patients (**Table S2**).

**Table 1:** Demographic characteristics in non-cachectic and cachectic cancer patients.

Variable	Cancer patients			F/χ^2^
	Total	Non-cachectic	Cachectic	
Number of patients	203	94	109	
**Age in years**				
Mean ± SD	51.45 ± 9.7	52.0 ± 8.8	50.98 ± 10.4	F = 2.9
Median (Range)	51 (20–77)	52 (30–77)	50 (20–75)	p = 0.46
**Gender [n (%)]**				
Male	107 (53)	45 (48)	62 (57)	**χ^2^** = 1.6
Female	96 (47)	49 (52)	47 (43)	p = 0.208
**Cancer type [n (%)]**				
Pancreatic cancer	145 (71)	76 (81)	69 (63)	**χ^2^** = 7.6
NSCL cancer	58 (29)	18 (19)	40 (37)	p = 0.008**
**Comorbidities [n (%)]**				
Negative	100 (49)	50 (54)	50 (46)	**χ^2^** = 1.2
Positive	103 (51)	44 (46)	59 (54)	p = 0.32
**No. of comorbidities [n (%)]**				
One	77 (75)	34 (77)	43 (73)	**χ^2^** = 0.26
>One	26 (25)	10 (23)	16 (27)	p = 0.65
**Type of chemotherapy [n (%)]**				
Xeloda	145 (71)	76 (81)	69 (63)	**χ^2^** = 7.6
Gem/Cis	58 (29)	18 (19)	40 (37)	p = 0.008**

Data are given as mean ± SD, median (minimum–maximum), or n (%). Statistical analysis was carried out using the independent t-test and chi-square test; p ≤ 0.05.

Cis, cisplatin; F, independent t-test value; Gem, gemcitabine; n (%), number of cases within the group (percentage); NSCL, non-small cell lung; χ^2^, chi-square value.

**Significant difference at p ≤ 0.01.

### Distribution of *TNF-α 308G/A* or *−1031T/C* Polymorphisms and Genotypes Among Pancreatic and Non-Small Cell Lung Cancer Patients

**Figure 1:**
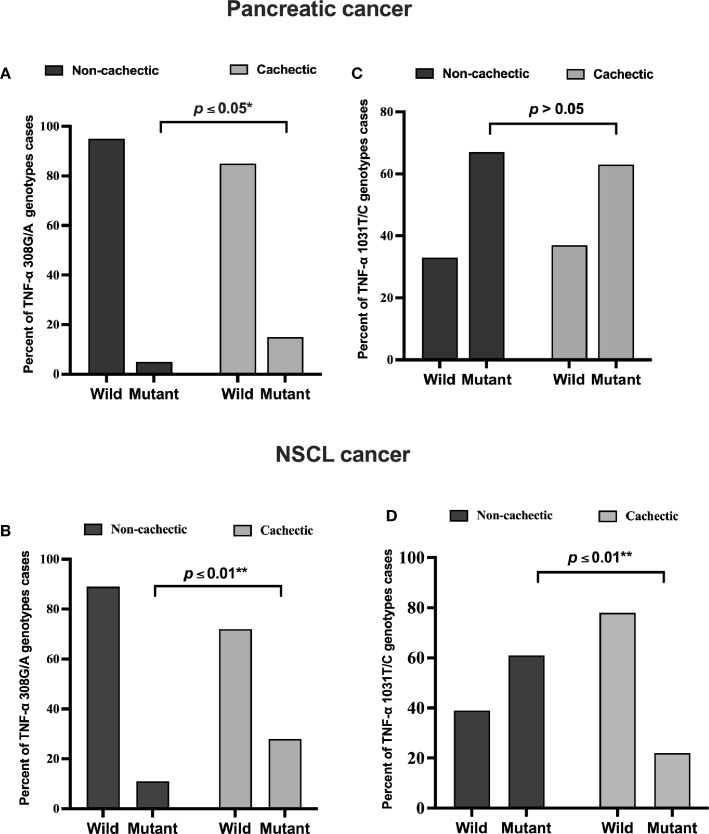
Frequencies of *TNF-α 308 G/A* (rs1800629) wild and mutant alleles among **(A)** pancreatic **(B)** NSCL cancer patients and *TNF-α T/C* 1031 (rs1799964) wild and mutant alleles among **(C)** pancreatic and **(D)** NSCL cancer patients. Comparison between wild and mutant alleles as performed by chi-square test at *p < 0.05. **Significant difference at p ≤ 0.01. NSCL, non-small cell lung.

In the pancreatic cancer group, 69 out of 145 patients were cachectic, whereas in the NSCL cancer group, 40 out of 58 were cachectic. According to the present allele frequency distribution data of all cancer patients, only 27 patients of 203 (13%) had the *TNF-α 308G/A* (rs1800629) mutation, whereas approximately seven times more patients carried the wild-type allele (176 patients, i.e., 87% of the total number). Of the 14 pancreatic cancer patients carrying the mutant *TNF-α 308G/A* (rs1800629) gene, 10 patients were cachectic (15%), and four patients were non-cachectic (5%), showing a significant positive association between *TNF-α 308G/A* gene mutation and cachexia (**Figure 1A**). In the NSCL cancer group, in a total of 13 patients, 11 patients who were cachectic (28%) and only two patients who were non-cachectic (11%) carried this allele, revealing also a significant positive association (**Figure 1B**). No significant association was observed between the polymorphism of *TNF-α 1031T/C* (rs1799964) in pancreatic cancer subgroups (**Figure 1C**), whereas a significant negative association (**Figure 1D**) was found between *TNF-α 1031T/C* gene mutation and cachexia in the NSCL cancer group. As expected, for an admixed population from Egypt, the frequency of the *TNF-α 308G/A* (χ^2^: 5.2, p = 0.001) and *TNF-α 1031T/C* (χ^2^: 3.8, p = 0.15) gene polymorphisms were higher than in Africans, Latin Americans, Asians, and Europeans (**Table S3**).

**Figure 2:**
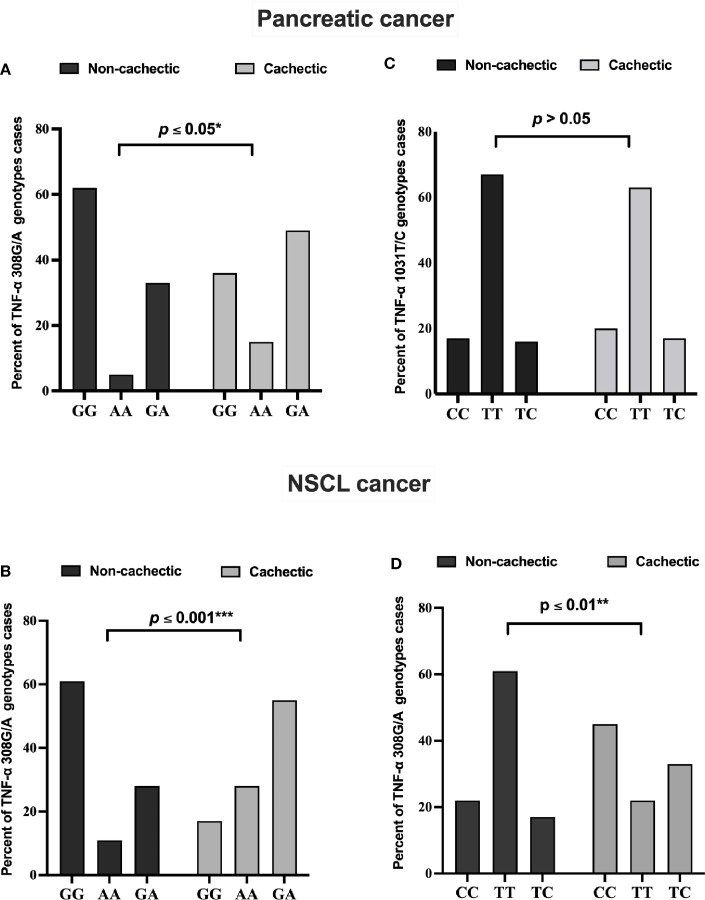
Frequencies of *TNF-α 308 G/A* genotypes among **(A)** pancreatic and **(B)** NSCL cancer patients and *TNF-α 1031 T/C genotypes* among **(C)** pancreatic and **(D)** NSCL cancer patients. Comparison between all genotypes was performed by chi-square test at *p ≤ 0.05. *Significant difference at p ≤ 0.05. **Significant difference at p ≤ 0.01 and *** at p ≤ 0.001. NSCL, non-small cell lung.

The genotype distribution of *TNF-α 308G/A* polymorphism in each cancer type is presented in **Figure 2**. In the cachectic pancreatic cancer, the heterozygous GA genotype (49%) showed the highest frequency among the three, whereas the homozygous GG genotype (62%) was the most frequently distributed among the non-cachectic group; and the homozygous AA genotype was more frequently distributed in cachectic (15%) than non-cachectic (5%) patients (**Figure 2A**). In the cachectic NSCL cancer patients, the heterozygous GA genotype (55%) showed the highest frequency among the three, whereas the homozygous GG genotype (61%) was the most frequently distributed among the non-cachectic group; and the homozygous AA genotype was more frequently distributed in cachectic (28%) compared with non-cachectic (11%) patients (**Figure 2B**). Similarly the genotype distribution of *TNF-α 1031T/C* is presented also in **Figure 2**. However, the *TNF-α 1031T/C* genotype was not associated with cancer-related cachexia in pancreatic cancer patients (**Figure 2C**). In contrast, in the cachectic NSCL cancer patients, the homozygous CC genotype (45%) showed the highest frequency among the three, whereas the homozygous TT genotype (61%) was the most frequently distributed among the non-cachectic group; and the heterozygous TC genotype was more frequently distributed in cachectic (33%) than non-cachectic (17%) patients (**Figure 2D**).

### Impact of *TNF-α 308G/A* or *−1031T/C* Gene Mutation Frequency on Cachexia Severity Score Among Pancreatic and/or Non-Small Cell Lung Cancer Patients

In both pancreatic (**Tables 2** and **S4**) and NSCL (**Tables 3** and **S5**) cancer patients, no significant association existed between *TNF-α 308G/A* or *TNF-α 1031T/C* mutant alleles as well as their allelic genotypes and the severity of cachexia. Similarly, no significant association was reached between *TNF-α 308G/A* or *TNF-α 1031T/C* allelic genotypes and the cachexia severity, regardless of the cancer type (**Table S6**).

**Table 2:** Distribution of *TNF-α* gene alleles among cachectic pancreatic cancer patients considering the cachexia severity.

Variable	*TNF-α 308G/A* (rs1800629)		*TNF-α 1031T/C* (rs1799964)	
	Wild	Mutant	Wild	Mutant
**Cachexia severity**				
** Pre-cachexia** (n = 20)	17 (85)	3 (15)	8 (40)	12 (60)
** Cachexia** (n = 32)	29 (91)	3 (9)	10 (31)	22 (69)
** Refractory** (n = 17)	13 (77)	4 (23)	8 (47)	9 (53)
** χ^2^**	1.8 p = 0.42		0.19 p = 0.90	

Data are given as n (%). Statistical analysis was carried out using the chi-square test; p ≤ 0.05.

n (%), number (percentage); rs, referred sequence; TNF-α, tumor necrosis alpha subunit gene; χ^2^, chi-square value.

**Table 3:** Distribution of *TNF-α* gene genotype among cachectic NSCL cancer patients considering the cachexia severity.

Variable	*TNF-α 308G/A* (rs1800629)		*TNF-α 1031T/C* (rs1799964)	
	Wild	Mutant	Wild	Mutant
**Cachexia severity**				
** Pre-cachexia** (n = 10)	9 (75)	3 (25)	7 (70)	3 (30)
** Cachexia** (n = 23)	8 (67)	4 (33)	20 (87)	3 (13)
** Refractory** (n = 7)	12 (75)	4 (25)	4 (57)	3 (43)
** χ^2^**	2.0 p = 0.38		3.2 p = 0.2	

Data are given as n (%). Statistical analysis was carried out using the chi-square test; p ≤ 0.05.

n (%), number (percentage); rs, referred sequence; TNF-α, tumor necrosis alpha subunit gene; χ^2^, chi-square value; NSCL, non-small cell lung.

### Impact of *TNF-α 308G/A* or *−1031T/C* Single-Nucleotide Polymorphisms on Susceptibility to Cancer-Associated Cachexia

After correction for multiple comparisons, both rs1799964 and rs1800629 showed a significant association with cachexia regardless of cancer type. In the unconditional logistic regression analysis, individuals with *TNF-α 308G/A* mutant genotypes had a significantly increased risk of cachexia as compared with those with the wild genotype. On the other hand, individuals with *TNF-α −1031T/C* mutant genotypes had a significantly decreased risk of cachexia as compared with those with the wild genotype. Moreover, the dominant and recessive models were analyzed, and the genotypic models for both SNPs were tested as follows (GG vs. GA and AA) for the SNP rs1800629 and (CC versus TC and TT) for the rs1799964: significant associations with both pancreatic cancer and NSCL cancer-associated cachexia were reached (**Table 4**).

**Table 4:** Risk factors for cachexia associated with cancer by binary logistic regression analysis.

Risk factor	β^0^	p-Value	Odds ratio	95% CI for Exp(B)
**TNF-α 308G/A, rs1800629**				
*Wild/mutant* regardless of cancer type	−0.355	0.04*	0.701	0.28–1.705
*Wild/mutant* for pancreatic cancer	−0.215	0.014*	0.414	0.203–0.845
*Wild/mutant* for NSCL cancer	−0.548	0.0001***	0.5	0.345–0.724
**TNF-α 1031T/C, rs1799964**				
*Wild/mutant* regardless of cancer type	0.706	0.02^*^	2.02	1.12–3.673
*Wild/mutant* for pancreatic cancer	0.881	0.012*	1.605	1.07–2.39
*Wild/mutant* for NSCLC	0.916	0.004**	2.5	1.67–751

For rs1800629, GA and AA are coded as 0, while GG carriers are coded as 1. For rs1799964, TC and TT are coded as 0, while CC carriers are coded as 1. Data are presented as odd ratio and 95% CI. p ≤ 0.05.

*Significant difference at p ≤ 0.05.

**Significant difference at p ≤ 0.01.

***Significant difference at p ≤ 0.001.

### Serum *MiRNA-155* Is Associated With Cachexia and Its Severity in Patients With Pancreatic or Non-Small Cell Lung Cancer

**Figure 3:**
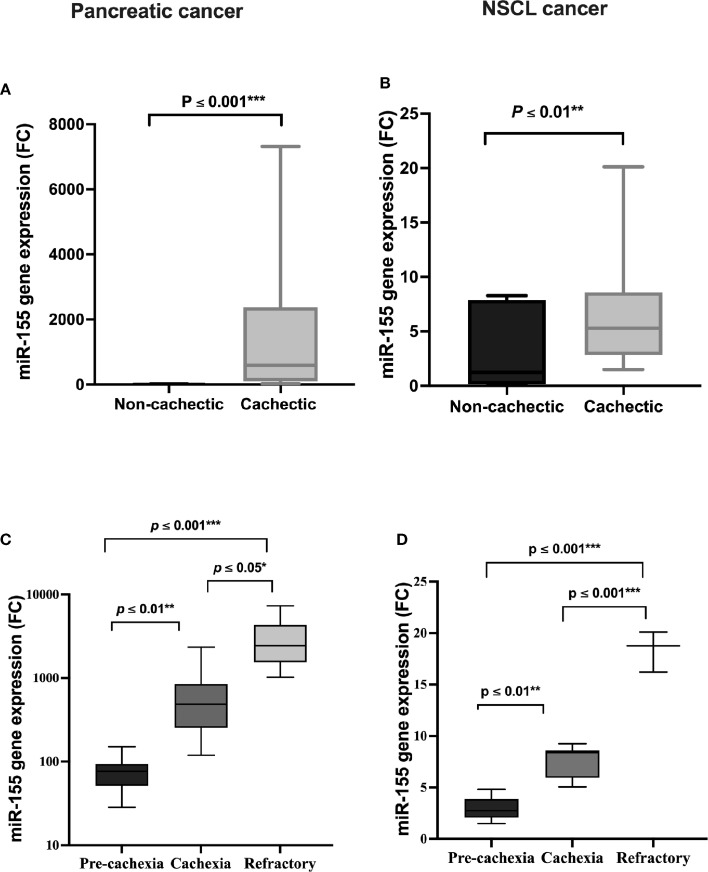
Serum expression of *miR-155* in **(A)** pancreatic and **(B)** NSCL non-cachectic and cachectic cancer patients and its expression in different grades of cachexia severity in **(C)** pancreatic and **(D)** NSCL cancer patients. Comparison between cachectic and non-cachectic groups was performed by chi-square test at *p < 0.05. **Significant difference at p ≤ 0.01 and *** at p ≤ 0.001. Comparison between all cachexia grades was performed by ANOVA test followed by Bonferroni’s post-hoc test; at *p < 0.05. **Significant difference at p ≤ 0.01 and *** at p ≤ 0.001. NSCL, non-small cell lung.

Circulating miRNAs have recently emerged in cancer cachexia and are a promising class of biomarkers. In the present study, a significant positive association of serum *miR-155* expression level between cachectic and non-cachectic patients existed, where *miR-155* is increased by 424-fold in the cachectic patients compared with the non-cachectic group in pancreatic cancer (**Figure 3A**); and also in NSCL cancer, *miR-155* was upregulated to 4.5-fold with cancer-associated cachexia (**Figure 3B**). This means that patients with cancer who have high *miR-155* have an increased likelihood of developing cancer cachexia than those with low *miR-155*. Overexpression of *miR-155* was even higher in cachectic patients with increasing phases (pre-cachexia, cachexia, refractory cachexia) in both pancreatic (**Figure 3C**) and NSCL (**Figure 3D**) cancers.

### Serum Level of SOCS1, TAB2, and Foxp3 in Patients With Pancreatic and Non-Small Cell Lung Cancer

In pancreatic cancer patients, a significant negative association was recorded between serum SOCS1 and Foxp3 with the presence of cachexia. Lower levels of SOCS1 (**Table 5**) and Foxp3 (**Table 5**) were observed in the cachectic group as compared with the non-cachectic one. On the other hand, there was no association between TAB2 and the presence of cachexia (**Table 5**). Regarding the association between the SOCS1, TAB2, and Foxp3 with the severity of cachexia, no significant association was detected except for Foxp3, where lower levels were significantly associated with higher severity of cachexia in patients with pancreatic cancer (**Table 5**).

**Table 5:** Serum SOCS1, TAB2, and Foxp3 level in cachectic and non-cachectic pancreatic and NSCL cancer patients and cachexia severity.

Variable	SOCS1 (ng/ml)	TAB2 (ng/ml)	Foxp3 (ng/ml)
**Pancreatic cancer patients**					
** Non-cachectic** (n = 20)	9.3 (7–26)	6.3 (2.0–8.4)	16 (5–30)
** Cachectic** (n = 48)	5.6 (3.3–8.6)	6.2 (4.8–13.0)	8.9 (6.5–96.0)
**Statistics**	U: 185 p = 0.002**	U: 262 p = 0.06	U: 49 p = 0.0001***
**Cachexia severity**
** Pre-cachexia** (n = 14)	5.6 (3.2–6.5)	6.5 (5.2–8.4)	16 (7–35)
** Cachexia** (n = 17)	5.2 (4.2–8.6)	6.3 (5.9–8.4)	14.5 (6–33)
** Refractory** (n = 17)	5.8 (3.7–7.4)	5.6 (2.6–7.8)	11.3 (5.6–30)
**Statistics**	F: 1.1 p = 0.34	F: 0.2 p = 0.8	F: 5.3 p = 0.008**
**NSCL cancer patients**					
** Non-cachectic** (n = 10)	10.9 (4.0–14)	9.2 (7.0–20)	8.8 (6.0.11)
** Cachectic** (n = 18)	5.8 (4.0–9.2)	8.0 (5.6 –29)	6.8 (4.6–9.7)
**Statistics**	U: 39 p = 0.01**	U: 57 p = 0.12	U: 35 p = 0.03*
**Cachexia severity score**			
** Pre-cachexia** (n = 9)	7.2 (4.2–9.2)	10 (5.6–29)	7.4 (4.6–9.7)
** Cachexia** (n = 7)	5.5 (4.5–6.8)	7.5 (7.0–9.0)	6.9 (4.9–7.8)
** Refractory** (n = 2)	5.2 (4.8–5.4)	6.4 (6.0–6.8)	NA
**Statistics**	F: 2.3 p = 0.12	F: 1.2 p = 0.34	F: 1.2 p = 0.33

Data are given as median (minimum–maximum). Statistical analysis was carried out using the Mann–Whitney test and ANOVA test followed by Bonferroni’s post-hoc test; p ≤ 0.05.

F, ANOVA test value; Foxp3, forkhead box P3; n, number; SOCS1, suppressor of cytokine signaling 1; TAB2, TAK1-associated Binding Protein 2; U, Mann-Whitney; NSCL, non-small cell lung.

*Significant difference at p < 0.05.

**Significant difference at p ≤ 0.01.

***Significant difference at p ≤ 0.001.

Regarding the NSCL cancer patients, significantly lower serum levels of SOCS1 and Foxp3 were noted in cachexia as compared with non-cachexia. The median serum level for SOCS1 was 10.9 in the non-cachectic patients and nearly half this value at 5.8 in cachectic patients (**Table 5**). Similarly, Foxp3 was 8.8 in the non-cachectic patients compared with 6.8 in the cachectic patients (**Table 5**). In contrast, no significant association was detected between the TAB2 and the presence of cachexia in the NSCL cancer patients (**Table 5**). Moreover, there was no association between SOCS1, TAB2, and Foxp3 with the cachexia severity in NSCL cancer (**Table 5**).

### SOCS1 Correlates Positively With Foxp3 in Cachexia Associated With Pancreatic Cancer

Correlation analyses (**Table 6**) revealed a strong positive correlation between SOCS1 and Foxp3 in the cachectic pancreatic cancer patients. On the other hand, no significant correlation was detected between serum *miR-155* and any of the targeted proteins (SOCS1, TAB2, and Foxp3) or between TAB2 and Foxp3 levels. Similarly, for the NSCL cancer patients with cachexia, no significant correlation was detected among the serum *miR-155* and any of the three targeted proteins (**Table 6**).

**Table 6:** Correlation analysis between *miR-155* gene expression and SOCS1, TAB2, and Foxp3 in cachectic pancreatic or NSCL cancer patients (Spearman’s correlation).

	*miR-155* (FC)	SOCS1 (ng/ml)	TAB2 (ng/ml)
**Cachectic pancreatic cancer patients**			
** miR-155 (FC)**			
** SOCS1 (ng/ml)**	0.17 p = 0.3		
** TAB2 (ng/ml)**	0.002 p = 0.9	−0.17 p = 0.2	
** Foxp3 (ng/ml)**	0.1 p = 0.56	0.69 p = 0.001***	0.08 p = 0.4
**Cachectic NSCL cancer patients**			
** miR-155 (FC)**			
** SOCS1 (ng/ml)**	−0.3 p = 0.2		
** TAB2 (ng/ml)**	−0.4 p = 0.09	−0.2 p = 0.1	
** Foxp3 (ng/ml)**	−0.2 p = 0.3	−0.2 p = 0.6	0.08 p = 0.4

Data are given as *r*. Statistical analysis was carried out using Spearman’s correlation analysis; p ≤ 0.05.

FC, fold change; Foxp3, forkhead box P3; *miR-155*, microRNA-155; n, number; *r*, Spearman’s correlation coefficient; SOCS1, suppressor of cytokine signaling 1; TAB2, TAK1-associated Binding Protein 2; NSCL, non-small cell lung.

***Significant difference at p ≤ 0.001.

### Serum *MiRNA-155* Is Associated With the Severity of Cachexia in Cancer Patients Regardless of the Type of Cancer But Not With SOCS1, TAB2, and Foxp3

A significant positive association was detected between serum *miR-155* level and the severity of cachexia in cancer patients regardless of the type of cancer where *miR-155* was increased by approximately sixfold in patients with refractory cachexia compared with cachectic patients, and it was also upregulated to around ninefold in cachectic patients compared with pre-cachectic patients (**Table 7**). In contrast, the severity of cachexia was not associated with the expression of SOCS1, TAB2, and Foxp3 (**Table 7**).

**Table 7:** Expression level of miR-155, SOCS1, TAB2, and Foxp3 in cancer cachectic patients considering the cachexia severity, regardless of the cancer type.

Cachexia severity	*MiR-155 *(FC)	SOCS1 (ng/ml)	TAB2 (ng/ml)	Foxp3 (ng/ml)
**Pre-cachexia**	46.2 (1.4–151)	5.8 (3.3–9.2)	10 (5.6–29)	6.3 (5.2–8.4)
**Cachexia**	431 (5.1–2348)	5.4 (4.2–8.6)	12 (7–25)	6.6 (4.8–8.4)
**Refractory**	2688 (16.5–7316)	5.8 (3.7–7.4)	16 (5.2–30)	6 (2.6–10)
**Statistics**	F: 29 p = 0.0001***	F: 1.2 p = 0.3	F: 0.2 p = 0.8	F: 1.3 p = 0.3

Data are given as median (minimum–maximum). Statistical analysis was carried out using the ANOVA test followed by Bonferroni’s post-hoc test; p ≤ 0.05.

F, ANOVA test value; FC, fold change; Foxp3, forkhead box P3; *miR-155*, microRNA-155; SOCS1, suppressor of cytokine signaling 1; TAB2, TAK1-associated Binding Protein 2.

***Significant difference at p ≤ 0.001.

## Discussion

Cancer cachexia is a polygenic complex syndrome in which a dysregulated inflammatory response partakes in its development (32). In this study, we first identified the genetic variants of *TNF-α 308G/A* (rs1800629) and *TNF-α 1031T/C* (rs1799964) and their association with cachexia in pancreatic and lung cancer Egyptian patients. To the best of the authors’ knowledge, the genotypic and allelic associations of *TNF-α 308G/A* and *TNF-α 1031T/C* gene polymorphisms with cachexia in pancreatic and NSCL cancers have not been unveiled before, especially in the Egyptian population. *TNF-α 308G/A* (rs1800629) gene polymorphism was a significant predictor for cachexia in both the lung and pancreatic cancer patients rather than that of *TNF-α 1031T/C* (rs1799964) gene, which was associated with lower risk of cachexia in the NSCL cancer patients. Of note, the homozygous GG genotype (wild) was mainly distributed among the non-cachectic group, and the homozygous AA (mutant) genotype was more frequently distributed in the cachectic than non-cachectic patients with pancreatic cancer. Regarding *TNF-α 308G/A* gene polymorphism in the NSCL cancer group, the heterozygous GA genotype was frequently detected in patients of the cachectic group, followed by the homozygous AA genotype with the least percent carrying the homozygous GG genotype. Secondly, we attempted to examine the involvement of *miR-155*/SOCS1/Foxp3/*TNF-α* signaling and *TAB2* in the pathogenesis of cachectic cancer patients. In this context, higher serum *miR-155* expressions were correlated with susceptibility to cachexia and were in parallel with its severity in both cancer types. Meanwhile, lower protein expression of SOCS1 and Foxp3 was only evident in both cachectic cancer groups without any association between TAB2 protein expression and the presence of cachexia. Moreover, lower protein expression of Foxp3 was significantly associated with higher severity of cachexia in patients with pancreatic cancer.

Cancer cachexia is a devastating multifactorial and often irreversible syndrome that affects approximately 50%–80% of cancer patients, depending on tumor type. It leads to substantial weight loss, primarily from loss of skeletal muscle and body fat (32, 33). Genetic variations are likely to contribute to the susceptibility or resistance to developing cancer cachexia. The role of TNF-α, one of the important genetic variants of genes encoding pro-inflammatory cytokines, has been reported in cancer cachexia (34). More than 100 gene variants that are linked to development of cachexia in cancer patients have been identified (35). SNPs, the most common type of heritable and evolutionarily stable genetic variations in the population, seem to be an attractive option for the selection of patients with high risk of cachexia (35). The *TNF-α 308 G/A* polymorphisms is one of the most frequently related with risk of malnutrition and tumor aggressiveness (33). A study on Tunisian population demonstrated a positive association between the TNF-α (-308 G/A) polymorphism and breast cancer susceptibility (36). Results of the study of Ahmad et al. (37) among an Indian population, suggest that TNF-α-308G/A polymorphism showed significant association with breast cancer patients (37). Our results show that the mutant *TNF-α* variant of *308 G/A* was significantly associated with increased risk of cachexia in both the pancreatic and NSCL cancer patients. On the contrary, that of 1031T/C was significantly associated with reduced risk of cachexia in the NSCL cancer patients. Notably, Barber et al. (38) have demonstrated that the A allele positivity in 308 gene loci confers approximately to a 3.0-fold increased susceptibility to malnutrition and cachexia in patients with end-stage renal disease (38), to consolidate the present data regarding *TNF-α 308G/A* gene polymorphism in both the pancreatic and NSCL cancer patients. Actually, the findings of the present study showed that the heterozygous GA genotype was detected in 55% of the lung cancer patients of the cachectic group, followed by the homozygous AA genotype (28%) with only 17% carrying the homozygous GG genotype. As for *TNF-α 308G/A* gene polymorphism in pancreatic cancer patients, the heterozygous GA genotype was frequently distributed (49%) in the cachectic group, whereas the wild homozygous GG genotype was frequently distributed (62%) among the non-cachectic group, and the homozygous AA genotype was more frequently distributed in cachectic (15%) than non-cachectic (5%) patients with pancreatic cancer.

Few data are available regarding the association of *TNF-α −1031T/C* genotype variant with cancer-related cachexia or inflammation. Nourian et al. (39), one of the recent studies, studying the role of genetics in Iranian patients with inflammatory bowel diseases (IBDs), reported that CC haplotype was associated with genetic risk of IBD (14). A previous study, however, found no association between the TNF-α polymorphisms at position −1031 and susceptibility to IBD (39). Moreover, in a study conducted on head and neck cancer patients, Powrózek et al. (35), investigating the potential role of *TNF-α 1031T/C* SNP as a risk factor for cachexia after radiotherapy, demonstrated that the C allele represents the unfavorable allele that is significantly associated with higher risk of cachexia, lower BMI, and shorter overall survival as compared with the TT or TC genotype carriers. Besides, the CC genotype carriers had a 9.7- to 13.2-fold higher risk of cachexia with the highest level of plasma TNF-α that directly reflects the alternation in patients’ nutritional status due to the underlying inflammatory response (35). In alignment, our results revealed that in the NSCL cancer patients, the homozygous CC genotype of *TNF-α 1031T/C* constitutes 45% of the cachectic patients, 33% for the heterozygous TC, and 22% for the homozygous TT genotype, contrary to pancreatic cancer patients where the homozygous TT is the most frequent genotype constituting 63% of the cachectic patients, followed by the homozygous CC genotype constituting 20% of cachectic patients and 17% for the heterozygous TC.

Our finding on the frequency of genetic polymorphisms in Egyptian pancreatic and NSCL cancer patients reported herein does not match that of Africans, Latin Americans, Asians, or Europeans (**Table S3**). This supports previous reported data related to Egyptians and non-Egyptians (40–43). Indeed, previous studies also revealed such a discrepancy among Asian and non-Asian ethnicity regarding the TNF-308 G/A polymorphisms in hepatocellular carcinoma risk (12). This could be explained by different factors attributed to the unmatched ethnic population and different pathological nature of the disease.

Skeletal muscle metabolism plays a crucial role in the pathogenesis of cachexia in cancer patients (44), where *miRNAs* are abundantly expressed in skeletal muscles and are involved in cancer cachexia. Numerous *miRNAs* are known to modulate skeletal muscle and adipose tissue turnover; therefore, the potential of *miRNAs* as predictor biomarkers and their clinical relevance in cachexia have been previously suggested (45). Indeed, their aberrant expression is associated with impaired myogenesis, consequently promoting the development of cachexia (13, 46). *MiRNAs* are also involved in the pathogenesis of different diseases including cancers and autoimmune diseases (47). *MiR-155* gene was found to be overexpressed in several solid tumors, such as thyroid carcinoma as well as breast and colon cancer (21). Moreover, altered *miRNA* expression has been found in several types of lymphoma and leukemia (19), and their role in cancer-associated cachexia has been earlier documented (47). In the current study, the levels of *miR-155* were significantly higher in the cachectic groups as compared with the non-cachectic groups, which was in alignment with the cachexia severity in both the pancreatic and NSCL cancer patients. This is in accordance with the observations that higher expression of *miR-155* was significantly associated with cancer progression and accelerates the development of cachexia in breast cancer patients (32, 48, 49). Consistent with our results, Wu et al. (50) have demonstrated that tumor-originated exosomal *miR-155* promotes the differentiation and remodels the metabolism of adipocytes in breast cancer (50).

The findings of the present study show lower levels of SOCS1 and Foxp3 together with higher expression of *miR-155* in the cachectic patients of both pancreatic and NSCL cancers in contrast to non-cachectic patients. Of note, the oncogenic role of *miR-155* in several cancer types has been previously addressed (51, 52). Regarding signaling pathway of *miR-155*, SOCS1 has been identified as a direct functional target of miR-155 (53) by enhancing TNF-α expression *via* SOCS1 suppression (54), hence elevating *TNF-α* cellular levels (55, 56). Therefore, these data delineate SOCS1 reduction in cachectic cancer patients in the present study.

Since *miRNAs* can often feedback to inhibit the transcription factor required for its induction (57), they might function as important epigenetic switches required for the functional maintenance of the cell type (58, 59). In this context, Foxp3, a transcription factor that is required for the maintenance of regulatory T cells (Treg), was shown to drive the high level of *miR-155* expression found in these cells to be followed by *miR-155*-mediated feedback inhibition of its target Foxp3 (58) *via* an *indirect* mechanism (60). This can afford a reasonable explanation for the low levels of Foxp3 with a high expression of *miRNA-155* in cachectic patients of the present study. Additionally, Foxp3 serum level indirectly correlates with cachexia severity only in the pancreatic cancer patients. Such an effect is in alignment with Gerriets et al. (61) who showed that conditions such as inflammation resulting from cachexia provide signals that increase glycolysis and expression of glucose transporter 1 (Glut1) levels in Treg. These metabolic changes directly modify Treg-cell function to downregulate the transcription factor Foxp3 (61). Moreover, there is a significant correlation among SOCS1 and Foxp3 protein in cachectic patients with pancreatic cancer. Similarly, the results of Collins et al. (62) results strongly suggest that SOCS1 contributes to the stability of the Foxp3^+^ Treg peripheral population under conditions of strong pro-inflammatory environments (62).

Apart from SOCS1 and Foxp3 involved in the oncogenic inflammatory machinery, TAB2 is a signaling molecule downstream of TNF receptor-associated factor 6 (TRAF6) that activates MAPKs (63). Intriguingly, Ceppi et al. (64) supported that TAB2 is considered a direct protein target of *miRNA*s in TLR signaling pathway (64). On the contrary, our results showed no significant association between the TAB2 protein and the presence of higher serum levels of *miR-155* or cachexia in both cancer groups. Since SOCS1, Foxp3, and TAB2 are components of several other TLR signaling pathways, hence, once one TLR is triggered, *miRNA*-mediated targeting of common signaling proteins could silence signaling through multiple TLRs (54, 58).

The authors are aware that the study was conducted on small scale of population that represents the main limitation. Another limitation was the lack of non-treated groups; the current study was also not longitudinal, and it was therefore not possible to follow up the progression of cachexia in the patients. Despite these limitations, this case study shows that carriers of the A allele *308 G/A* gene and high *miR-155* are at greater risk of cachexia in both the pancreatic and NSCL cancer patients; however, the mutant variant of *1031T/C* gene is protective against cachexia in the NSCL cancer patients. Nonetheless, further studies should be carried out on the two *TNF-α* SNPs on larger scale of patients in order to confirm their predictive/prognostic significance. Finally, high levels of *miR-155* in the cachectic group lead to negative feedback inhibition of both SOCS1 and Foxp3 in both the pancreatic and NSCL cancer patients.

## Data Availability Statement

The datasets presented in this study can be found in online repositories. The names of the repository/repositories and accession number(s) can be found in the article/**Supplementary Material**.

## Ethics Statement

The studies involving human participants were reviewed and approved by Research Ethics Committee of Faculty of Pharmacy, Cairo University (Cairo, Egypt; PT-2387) as well as the Ethical Committee of the Oncology department, Ain Shams University. The patients/participants provided their written informed consent to participate in this study.

## Author Contributions

RY and AS performed the research. SS, MS, and DA wrote the research. SS, MS, DA, and AS designed the research. RY, MS, SS, DA, and NS analyzed the data. All authors contributed to the article and approved the submitted version.

## Conflict of Interest

The authors declare that the research was conducted in the absence of any commercial or financial relationships that could be construed as a potential conflict of interest. The handling editor declared a shared affiliation with several of the authors AS and SS at time of review.

## Publisher’s Note

All claims expressed in this article are solely those of the authors and do not necessarily represent those of their affiliated organizations, or those of the publisher, the editors and the reviewers. Any product that may be evaluated in this article, or claim that may be made by its manufacturer, is not guaranteed or endorsed by the publisher.

## Abbreviations

Foxp3, Forkhead box P3; SOCS1, Suppressor of cytokine signaling 1; TAB2, TAK1-associated Binding Protein 2; *TNF-α*, Tumor necrosis alpha subunit gene.
