# Supplementary material for: Impact of TNF-α Gene Polymorphisms on Pancreatic and Non-Small Cell Lung Cancer-Induced Cachexia in Adult Egyptian Patients: A Focus on Pathogenic Trajectories
Source: Front Oncol. 2021 Nov 18;11:783231. doi: 10.3389/fonc.2021.783231 (PMC8651494; doi:10.3389/fonc.2021.783231)
Supplement: Supplementary file 1 [file Table_1.docx]

**Table S1. Biochemical and Clinicopathological features of pancreatic cancer patients**

Data are given as mean± SD, range (minimum-maximum), or n (%). Statistical analysis was carried out using the independent t-test and Chi-square test; p **≤** 0.05. ***Significant difference at p **≤** 0.001. (%): percentage of cases within the group; ALT: Alanine Transaminases; AST: Aspartate transaminases; BUN: blood urea nitrogen; Hb: hemoglobin; n: number of cases within the group; NSCL: non-small cell lung; SD: standard deviation; ꭓ^2^: Chi-square value

| Variable | **Pancreatic Cancer group**  **(n= 145)** | |  |
| --- | --- | --- | --- |
|  | **Non-cachectic**  **n=76** | **Cachectic**  **n=69** | ꭓ^2^ |
| **Age**  Mean ±SD  Range) | 51.54±8.6  30 – 68 | 50.02±11.0  20 – 70 | 1.0  p= 0.309 |
| **Gender n (%)**  Male  Female | 32(42)  44(58) | 38(55)  31(45) | 2.4  p= 0.136 |
| **Co-morbidities n (%)**  Negative  Positive | 39(51)  37(49) | 31(55)  38(45) | 0.6  p= 0.507 |
| **No of Co-morbidities n (%)**  One  More than one | 28(76)  9(24) | 29(76)  9(24) | 0.004  p= 0.990 |
| **BUN (mg/dl)**  Mean ±SD  Range | 13.49±3.54  3 – 21 | 15.90±4.64  4 – 31 | 11.9  p= 0.001** |
| **AST (IU/L)**  Mean ±SD  Range | 30.33±8.58  12 – 50 | 30.74±13.00  7 – 90 | 0.11  p= 0.738 |
| **ALT (IU/L)**  Mean ±SD  Range | 26.48±10.68  8 – 57 | 26.03±11.20  5 – 56 | 0.37  p= 0.542 |
| **Total serum bilirubin (mg/dl)** Mean ±SD  Range | 0.70±0.26  0.28 – 1.70 | 0.83±0.73  0.30 – 5.70 | 0.92  p= 0.336 |
| **Direct serum bilirubin** **(mg/dl)** Mean ±SD  Range | 0.15±0.06  0.01 – 0.40 | 0.18±0.18  0.10 – 1.50 | 0.006  p= 0.936 |
| **Hb(mg/dl)**  Mean ±SD  Range | 12.16±1.29  10 – 15.90 | 11.85±1.46  8.5 – 14 | 1.2  p= 0.27 |
| **Platelets (x 1000/µl)**  Mean ±SD  Range | 258.3±79.5  140 – 430 | 272.3±86.1  100 – 502 | 1.4  p= 0.23 |
| **Total leukocytic count (x1000/µl)**  Mean ±SD  Range | 5.91±1.77  3 – 11 | 6.64±2.42  3.0 – 18.40 | 4.2  p= 0.04* |

**Table S2. Biochemical and Clinicopathological features of NSCL cancer patients**

Data are given as mean± SD, range (minimum-maximum), or n (%). Statistical analysis was carried out using the independent t-test and Chi-square test; p **≤** 0.05. (%): percentage of cases within the group; ALT: Alanine Transaminases; AST: Aspartate transaminases; BUN: blood urea nitrogen; Hb: hemoglobin; n: number of cases within the group; NSCL: non-small cell lung; SD: standard deviation; ꭓ^2^: Chi-square value

| **Variable** | **NSCLC**  **group (n= 58)** | | ꭓ^2^ | |
| --- | --- | --- | --- | --- |
|  | **Non-cachectic**  **n=18** | **Cachectic**  **n=40** |  |  |
| **Age**  Mean ±SD  Range | 53±9.6  38 - 77 | 53.0±9.2  29 - 75 | 0.001  p= 1.0 | |
| **Gender n (%)**  Male  Female | 13(72)  5(28) | 24(60)  16(40) | 0.8  p=0.3 | |
| **Co-morbidities n (%)**  Negative  Positive | 11(61)  7(39) | 19(48)  21(52) | 0.9  p= 0.25 | |
| **No of Co-morbidities n(%)**  One  >one | 14(67)  7(33) | 6(86)  1(14) | 0.9  p= 0.32 | |
| **BUN (mg/dl)**  Mean ±SD  Range | 17.0±3.2  12 - 21 | 15.6±3.6  8 – 22 | 0.96  p= 0.33 | |
| **AST (IU/L)**  Mean ±SD  Range | 26±8.2  16 - 40 | 27±7.6  9 – 40 | 0.18  p= 0.67 | |
| **ALT (IU/L)**  Mean ±SD  Range | 24±8.9  8 - 40 | 24±8.5  6 – 40 | 0.84  p= 0.77 | |
| **Total serum bilirubin (mg/dl)**  Mean ±SD  Range | 0.6±0.3  0.3 – 1.2 | 0.7±0.3  0.3 – 1.3 | 0.54  p= 0.46 | |
| **Direct serum bilirubin(mg/dl)**  Mean ±SD  Range | 0.1±0.07  0.01 – 0.30 | 0.13±0.04  0.06 – 0.3 | 5.2  p= 0.02 * | |
| **Hb(mg/dl)**  Mean ±SD  Range | 12±1.4  10 – 15 | 12±1.2  9 – 14 | 0.003  p= 0.96 | |
| **Platelets (x 1000/µl)**  Mean ±SD  Range | 286.3±82  178 – 450 | 296±92  159 – 450 | 1.48  p= 0.22 | |
| **Total leukocytic count (x1000/µl)**  Mean ±SD  Range | 5.4±2.2  3 – 10 | 5.3±2.0  3.0 – 10 | 0.34  p= 0.56 | |

**Table S3.** **Minor allele frequencies of studied genetic polymorphisms among Egyptian patients compared to reported population frequencies**

Data are given as frequency, Statistical analysis was carried out using the Chi square test; p **≤** 0.05. ***Significant difference at p**≤**0.001.

*****Minor allele frequencies obtained from the 1000 Genome, Phase 3 data (<http://phase3browser.1000genomes.org/index.html>). † Minor allele frequency reported in Asian populations, as defined by the 1000 Genomes Project. rs: referred sequence; SNP: single nucleotide polymorphism; *TNF-α*: Tumor necrosis factor alpha

| SNP | Genotype | Egyptian (Present study) | African* | Latin American* | Asian† | European* | ꭓ^2^ |
| --- | --- | --- | --- | --- | --- | --- | --- |
| *TNF-α* 308G/A (rs1800629) | G>A | 0.34 | 0.12 | 0.13 | 0.0 | 0.16 | 5.2  p=0.001*** |
| *TNF-α* 1031T/C  (rs1799964) | C>T | 0.41 | 0.16 | 0.23 | 0.21 | 0.21 | 3.8  p=0.15 |

**Table S4. Distribution of *TNF-α* allelic genotypes among cachectic pancreatic cancer patients considering the cachexia severity.**

Data are given as n (%). Statistical analysis was carried out using the Chi square test; p **≤** 0.05. n (%): number (percentage); rs: referred sequence; TNF- α: Tumor necrosis alpha subunit gene; ꭓ^2^: Chi-Square value

| Group | *TNF-α 308G/A* (rs1800629) | | | *TNF-α 1031T/C* (rs1799964) | | |
| --- | --- | --- | --- | --- | --- | --- |
|  | **GG** | **AA** | **GA** | **CC** | **TT** | **TC** |
| **Cachexia severity**  Pre-cachexia (n=20)  Cachexia (n=32)  Refractory (n=17) | 6(30)  12(38)  7(41) | 3(15)  3(9)  4(24) | 11(55)  17(53)  6(35) | 5(25)  4(16)  5(20) | 12(60)  15(60)  16(67) | 3(15)  6(24)  3(13) |
| **Statistics** | ꭓ^2^: 0.33, p=0.98 | | | ꭓ^2^: 1.4, p=0.84 | | |

**Table S5. Distribution of TNF-α gene allelic genotypes among cachectic NSCL cancer patients considering the cachexia severity.**

Data are given as n (%). Statistical analysis was carried out using the Chi square test; p **≤** 0.05. n (%): number (percentage); rs: referred sequence; TNF- α: Tumor necrosis alpha subunit gene; ꭓ^2^: Chi-Square value

| **Group** | TNF-α 308G/A (rs1800629) | | | TNF-α 1031T/C (rs1799964) | | |
| --- | --- | --- | --- | --- | --- | --- |
|  | **GG** | **AA** | **GA** | **CC** | **TT** | **TC** |
| **Cachexia severity**  Pre-cachexia  Cachexia  Refractory | 3(20)  2(15)  2(17) | 5(33)  4(31)  2(17) | 7(47)  7(54)  8(66) | 4(40)  12(52)  2(29) | 3(30)  3(13)  3(42) | 3(30)  8(35)  2(29) |
| ꭓ^2^ | 2.5, p=0.65 | | | 3.4, p=0.49 | | |

**Table S6. Distribution of *TNF-α* gene allelic genotypes among cancer cachectic group considering the cachexia severity, regardless the cancer type**

Data are given as n (%). Statistical analysis was carried out using the Chi square test; p **≤** 0.05. n (%): number (percentage); rs: referred sequence; TNF- α: Tumor necrosis alpha subunit gene; ꭓ^2^: Chi-Square value

| Group | *TNF-α 308G/A* (rs1800629) | | | *TNF-α 1031T/C* (rs1799964) | | |
| --- | --- | --- | --- | --- | --- | --- |
|  | **GG** | **AA** | **GA** | **CC** | **TT** | **TC** |
| **Cachexia severity**  Pre-cachexia  Cachexia  Refractory | 9(26)  14(31)  9(31) | 8(23)  7(16)  6(21) | 18(51)  24(53)  14(48) | 10(33)  16(30)  6(24) | 14(47)  24(44)  14(56) | 6(20)  14(26)  5(20) |
| ꭓ^2^ | 1.0, p=0.9 | | | 0.6, p=0.9 | | |
